# The Relevance of Insomnia in the Diagnosis of Perinatal Depression: Validation of the Italian Version of the Insomnia Symptom Questionnaire

**DOI:** 10.3390/ijerph182312507

**Published:** 2021-11-27

**Authors:** Lavinia De Chiara, Cristina Mazza, Eleonora Ricci, Alexia Emilia Koukopoulos, Georgios D. Kotzalidis, Marco Bonito, Tommaso Callovini, Paolo Roma, Gloria Angeletti

**Affiliations:** 1 Department of Neurosciences, Mental Health, Sensory Functions (NESMOS), Sant’Andrea University Hospital, Faculty of Medicine and Psychology, Sapienza University of Rome, Via Grottarossa 1035-1039, 00189 Rome, Italy; lavinia.dechiara@uniroma1.it (L.D.C.); giorgio.kotzalidis@uniroma1.it (G.D.K.); gloria.angeletti@uniroma1.it (G.A.); 2 Department of Neuroscience, Imaging and Clinical Sciences, G. d’Annunzio University of Chieti-Pescara, Via dei Vestini 31, 66100 Chieti, Italy; cristina.mazza@unich.it (C.M.); eleonora.ricci@unich.it (E.R.); 3 Department of Neuroscience/Mental Health, UOC Psichiatria, Psicofarmacologia Clinica, Azienda Ospedaliera Universitaria Policlinico Umberto I, Viale Regina Elena, 328, 00161 Rome, Italy; alexia.koukopoulos@uniroma1.it; 4 Dipartimento Materno Infantile, San Pietro Fatebenefratelli Hospital, Via Cassia, 600, 00189 Rome, Italy; bonitomarco@libero.it; 5 Department of Medicine and Surgery, University of Milano Bicocca, Via Cadore 48, 20900 Monza, Italy; t.callovini@campus.unimib.it; 6 Department of Human Neurosciences, Sapienza University of Rome, Viale Regina Elena 334, 00161 Rome, Italy

**Keywords:** Insomnia Symptom Questionnaire, sleep disorders, perinatal period, internal consistency, convergent validity

## Abstract

Background. Sleep disorders are common in perinatal women and may underlie or trigger anxiety and depression. We aimed to translate and validate and evaluate the psychometric properties of the Italian version of the Insomnia Symptom Questionnaire (ISQ), in a sample of women during late pregnancy and 6-months postpartum according to the DSM-5 criteria. Methods. The ISQ was administered to 292 women prenatally along with other measures of sleep quality, depression, and anxiety, to examine its construct and convergent validity. Women were readministered the ISQ six months postdelivery to assess test–retest reliability. Women were divided into DSM-5 No-Insomnia (*N* = 253) and Insomnia (*N* = 39) groups. Results. The insomnia group had received more psychopharmacotherapy, had more psychiatric family history, increased rates of medically assisted reproduction, of past perinatal psychiatric disorders, and scored higher on almost all TEMPS-A dimensions, on the EPDS, HCL-32, PSQI, and on ISQ prenatally and postnatally. ISQ scores correlated with all scales, indicating adequate convergent and discriminant validity; furthermore, it showed antenatal–postnatal test–retest reliability, 97.5% diagnostic accuracy, 79.5% sensitivity, 94.9% specificity, 70.5% positive predictive power, and 92.8% negative predictive power. Conclusions. The ISQ is useful, valid, and reliable for assessing perinatal insomnia in Italian women. The Italian version showed equivalent properties to the original version.

## 1. Introduction

The DSM-5 [1] defined insomnia as dissatisfaction with sleep quantity or quality, associated with one or more of the following symptoms: difficulty initiating sleep, difficulty maintaining sleep, frequent awakenings, or problems returning to sleep after awakenings. Sleep difficulties occur despite adequate opportunity for sleep, at least 3 nights per week, for at least 3 months. The sleep disturbance causes clinically significant distress or impairment in social, occupational, educational, academic, behavioral, or other important areas of functioning. In contrast to the DSM-IV-TR [2], the DSM-5 makes no distinction between primary and comorbid insomnia, and dissatisfaction with sleep quantity or quality were included as prerequisites for diagnosing insomnia. When assessing sleep quality, the accuracy of the definition of sleep quality itself is a fundamental issue. The International Classification of Sleep Disorders-Third Edition (ICSD-3) criteria [3] are consistent with the DSM-5. The updated version of the DSM does not conflict with the former version for what concerns symptom identification and assigning the diagnosis.

Sleep disturbances are highly prevalent during pregnancy [4,5]. Their trajectories may affect pregnancy and birth outcomes [6,7]. A recent meta-analysis indicated that 45.7% of expectant mothers experienced poor sleep quality [8], and another meta-analysis focusing on insomnia, reported a general mean of 38.2% which peaked during the last trimester [9]. Women experience dramatic physical changes during the perinatal period; while many adapt well to being pregnant, some become severely distressed [10,11,12]. Physical discomfort during pregnancy may involve possible chronic sleep disruption and fragmentation [13]. Sleep deprivation may predict mental disorders [14]. Therefore, it appears that anxiety, depression, and sleep disorders are related bidirectionally, independently of which one is the initial trigger [15].

Insomnia during pregnancy is a risk factor for postpartum depressive symptoms [16,17,18,19]. Conversely, mothers with depression have a higher risk of developing sleep disturbance [20,21]. Perinatal depression, defined as a major depressive episode during pregnancy or within the first year postpartum, is the most common complication of childbirth and a major public health problem affecting all members of the family while too often escaping detection and treatment.

Recent studies highlight the critical role of screening, early diagnosis, and suitable insomnia treatment during pregnancy in reducing depressive symptoms [22]. The association between poor sleep and perinatal psychiatric disorders has important clinical implications [23]; pregnant women who suffer from poor sleep quality can be identified easily by midwives or obstetricians during routine prenatal checkups, thus potentiating mood disorder prevention [24]. Given women’s reluctance to take psychotropic medications during pregnancy [25], sleep protection as nonpharmacological means to prevent and reduce postpartum mental illness has been advocated [26].

There are currently several self-reported questionnaires available for assessing sleep quality and insomnia in the general population. The Pittsburgh Sleep Quality Index (PSQI) [27,28] and the Insomnia Severity Index (ISI) [29,30] are the most widely used tools in Italy. Few tools are available in Italian to assess the prevalence of insomnia during the perinatal period. The Insomnia Symptom Questionnaire (ISQ) [31] is a 13-item self-report instrument designed to identify insomnia and validated to recognize insomnia in pregnant women [20]. ISQ questions are based on DSM-IV-TR criteria for primary insomnia. The questionnaire is a short and cost-effective tool that can be quickly employed in large observational studies or in clinical practice.

During pregnancy, sleep quality assessment should be advised to guide possible preventative and therapeutic interventions [23]. The aim of the present study was to translate and validate the ISQ and evaluate the psychometric properties of its Italian version in a sample of women during late pregnancy and 6 months postpartum according to the DSM-5 criteria.

## 2. Materials and Methods

### 2.1. Research Setting

The study was developed in the context of a collaborative screening effort between the Gynaecology and Obstetrics unit of San Pietro Fatebenefratelli Hospital of Rome, Italy, and the Center for Prevention and Treatment of Women’s Mental Health Problems, Psychiatry Unit, Sapienza University, Faculty of Medicine and Psychology, Sant’Andrea Hospital, Rome, Italy.

### 2.2. Participants

We recruited 304 women at the Gynaecology and Obstetrics unit of San Pietro Fatebenefratelli Hospital in Rome, a large maternity unit, between July and December 2018 during their routine third-trimester screening. The women included in the study were screened once during their third trimester of pregnancy (T0) and again six months postpartum (T1). We recruited 39 women with DSM-5 insomnia. Exclusion criteria were age less than 18 years old, failure to provide free informed consent, and incomplete comprehension of the Italian language that prevented participants from completing the questionnaires. Participants with an incomplete ISQ were also excluded from the final analysis (*N* = 12). Antenatal participants who had consented to be contacted in the postnatal period were called by two trained psychologists of our Centre for Prevention and Treatment of Women’s Mental Health, 6 months following the birth of their baby, and invited to complete the questionnaires again through an online system (Google Form).

The final study sample comprised 292 women, aged 19–46 years (mean = 33.26, SD = 5.04); 95% of participants (*N*= 278) were in a stable relationship, most of them held a university degree (*N* = 158, 54.1%) and were employed (*N* = 230, 78.8%), 160 participants (54.8%) reported changes in sleep hours. The sample was split into Insomnia (*N* = 39) and a No-Insomnia samples (*N* = 253) according to whether they met or not DSM-5 criteria for insomnia. Descriptive statistics of the two subsamples are presented in Results and Table 1.

Participants provided written informed consent, in accordance with all applicable regulatory and Good Clinical Practice guidelines and in full respect of the Ethical Principles for Medical Research Involving Human Subjects, as adopted by the 18th World Medical Association General Assembly (WMA GA), Helsinki, Finland, June 1964, and subsequently amended by the 64th WMA GA, Fortaleza, Brazil, October 2013. It was approved by the local ethics committees (Boards of the Sant’Andrea Hospital, Rome and San Pietro Fatebenefratelli Hospital, Rome, the ethics committee of Lazio 1, San Camillo-Forlanini Hospital, Rome, Italy; 4 December 2017 Nr 2471/CE Lazio1).

## 3. Procedure and Measures

Screening tools were administered by psychologists of the Psychiatry Unit. Women were evaluated through a sociodemographic, clinical, and obstetric data collection sheet (Perinatal Interview; PI), the Pittsburgh Sleep Quality Index (PSQI), the Edinburgh Postnatal Depression Scale (EPDS), the Zung Self-Rating Anxiety Scale (SAS), the Hypomania CheckList-32 (HCL-32), the Temperament Evaluation of the Memphis, Pisa, Paris and San Diego-Autoquestionnaire (TEMPS-A), and the Insomnia Symptom Questionnaire (ISQ).

Insomnia was diagnosed according to standard diagnostic criteria at the time of the evaluation by two psychiatrists, sleep medicine specialists who were blind to the screening scores, using the Mini International Neuropsychiatric Interview Version 7 for DSM-5 [32].

Included measures for the screening evaluation were the following:

- **Perinatal Interview (PI)** is a paper-and-pencil questionnaire to collect sociodemographic and clinical information, allowing us to investigate predictive and protective factors for the development of psychiatric disorders. Besides place and date of birth, nationality, educational level, job, and marital status, the PI investigates habits (i.e., eating, drinking, and weight control), voluptuary substance use (including coffee, tobacco, and alcohol), physiological rhythms (i.e., time to go to sleep, waking time, and sleeping hours), past surgery, past and current pharmacological treatment, gynecological and obstetric history, focusing on the current and past pregnancies, past and current personal and family psychiatric history and any psychiatric treatment, stressful life events, partner and family/friends’ support during pregnancy, and partner data.
- **The Pittsburgh Sleep Quality Index** (PSQI) [27,28] is a retrospective self-report questionnaire that measures sleep quality and disturbances over the previous month. The PSQI assesses seven clinically derived components of subjective sleep quality: 1. *sleep quality,* 2. *sleep latency,* 3. *sleep duration,* 4. *habitual sleep efficiency,* 5. *sleep disturbance,* 6. *use of sleep medications,* and 7. *daytime dysfunction*. The PSQI yields a global score that represents the sum of the seven component scores that are rated on a 4-point Likert scale ranging from 0 to 3, where 3 reflects the negative extreme of the Likert scale. A global score of 5 or higher is considered as an indicator of prominent sleep disturbance in at least two components or of moderate difficulties in more than three components, distinguishing between “good” and “bad” sleepers. In the Italian validation study [28], the PSQI showed high internal consistency with a Cronbach’s alpha of 0.84.
- **The Edinburgh Postnatal Depression Scale** (EPDS) [33] is a 10-item self-report questionnaire administered to screen for depressive symptoms in both the antenatal and postnatal periods [34,35]. We used the recommended score of 13 or more that indicates probable major depression in postnatal Italian-speaking women [36]. In the Italian validation study, the EPDS showed good internal consistency with a Cronbach’s alpha of 0.79.
- **The Zung Self-Rating Anxiety Scale** (SAS) [37] is a 20-item self-report assessment tool built to measure state anxiety levels. Raw scores range from 20 to 80. The initial cutoff was 50 [38], but the best cutoff was later proposed to be 40 for clinical settings and 36 for screening purposes [39]. The instrument is suited to investigate anxiety disorders and showed strong correlations with other similar instruments [40,41]. In this study, we used the Italian version [42].
- **The Hypomania CheckList-32** (HCL-32) [43] is a 32-item self-rating questionnaire investigating the lifetime history of hypomanic symptoms. Individuals scoring ≥ 14 potentially have bipolar disorder/diathesis and should be carefully interviewed. The ideal cutoff point of the Italian version is 12, with a sensitivity of 0.85 and a specificity of 0.61 [44].
- **The Temperament Evaluation of the Memphis, Pisa, Paris and San Diego-Autoquestionnaire** (TEMPS-A) [45], is a 110 item yes-or-no self-report questionnaire designed to assess affective temperament in psychiatric and healthy subjects. It consists of five temperament traits, i.e., depressive (D), cyclothymic (C), hyperthymic (H), irritable (I), and anxious (A). The prevailing temperament is considered the one on which the completer obtains the higher score. We used the validated Italian version [46].
- **Insomnia Symptom Questionnaire** (ISQ) [31] is a 13-item self-report instrument designed to assess respondents’ perceptions about their daytime functioning, nighttime sleep, and identify insomnia. The ISQ items are based on DSM-IV criteria for primary insomnia [2] and are consistent with the American Academy of Sleep Medicine’s (AASM) Research Diagnostic Criteria (RDC) [47]. Items 1, 2, or 5 (example item: *During the past month did you have difficulties falling asleep?*) are used to determine the presence, frequency, and duration of sleep symptom criteria (example: *How long did the symptom last?;* example answer: *# weeks/months/years*) and are rated on a 6-point Likert scale, ranging from 0 (*never*) to 5 (*always/5–7 times a week*). Items 6–13 are used to identify significant daytime consequences of the sleep complaint (example item: *During the past month have your sleep difficulties affected your work?*) and are rated on a 5-point Likert scale, ranging from 0 (*not at all*) to 4 (*extremely*). The final outcome of the ISQ is obtained through a dichotomous response (*yes/no*) to the three sleep criteria (*sleep symptom criterion* items 1, 2, or 5; *duration criterion items* 1, 2, or 5; *daytime impairment criterion* items 6–13), which results in the “presence” (3 yes answer) or “absence” of insomnia. In the validation study [31], the ISQ obtained a Cronbach’s alpha of 0.89, indicating a high degree of internal consistency. In our sample, the ISQ obtained a Cronbach’s alpha of 0.92, showing comparable if not higher internal consistency.
  The Italian translation of the ISQ was carried out through a direct and reverse translation process [48]. Specifically, a bilingual Italian/English psychiatrist translated the ISQ from English to Italian. Subsequently, another bilingual Italian/English researcher back-translated the scale.

After discussing any differences between the two translations, the scale was back-translated by a native speaker researcher, unaware of previous translations. The Italian version of the ISQ includes 13 items, rated as in the original version.

## 4. Data Analyses

Descriptive statistics of the two samples were analyzed using the Chi-squared test for categorical variables and ANOVAs for continuous variables. The convergent validity of the Italian version of the ISQ has been assessed by conducting point-biserial correlations (r_pb_) between the ISQ and the PSQI global score. We also dichotomized the PSQI global score at two cutoffs (> 5 and > 10) reflecting the scores used in the original study [31]. Discriminant validity has been evaluated between the ISQ and EPDS, HCL-32, and SAS global scores. ISQ reliability was tested using Cronbach’s alpha and test-retest reliability was assessed by examining the correlation between the total ISQ score in the antenatal (T0) and postnatal (T1) period for a subsample of participants (*N* = 49) who completed the ISQ both antenatally and six months postpartum. ISQ accuracy, sensitivity, specificity, and negative and positive predictive power were also investigated. Furthermore, to determine the best cutoff score of the PSQI that optimally detected cases defined by a presence or absence of a DSM-5 insomnia diagnosis, the receiver operating characteristic (ROC) curve analysis was run.

The IBM SPSS-25 statistical package (IBM Inc., Armonk NY, USA, 2017) was used for all analyses.

## 5. Results

### 5.1. Descriptive Statistics

#### 5.1.1. No-Insomnia Sample

The No-Insomnia sample included 265 Italian-fluent adult women of the general population screened during their third trimester of pregnancy (T0). The final sample consisted of 253 women, aged 19–46 (*mean* = 33.13 years; *SD* = 5.11).

#### 5.1.2. Insomnia Sample

The Insomnia sample included 39 women, screened during their third trimester of pregnancy (T0). Participants were aged 22–42 (*mean* = 34.10 years; *SD* = 4.60).

Statistically significant differences were found between the *No-Insomnia* and *Insomnia Samples* on TEMPS-A Depressive (*F* (1248) = 41.885; *p* < 0.001), Cyclothymic (*F* (1248) = 20.226; *p* < 0.001), Irritable (*F* (1242) = 26.140; *p* < 0.001), Anxious (*F* (1242) = 43.933; *p* < 0.001), and TEMPS-A Prevailing Temperament (*F* (1245) = 4.943; *p* = 0.027), on EPDS (*F* (1289) = 52.439; *p* < 0.001), on SAS (*F* (1271) = 31.315; *p* < 0.001), on HCL-32 (*F* (1254) = 8.674; *p* = 0.004), and on PSQI Global Score (*F* (1276) = 144.850; *p* < 0.001). Furthermore, statistically significant differences were found between the No-Insomnia and Insomnia Samples on Previous Psychopharmacological Therapy (*χ*^2^ = 16.772; *p* < 0.001), Psychiatric Family History (*χ*^2^ = 10.287; *p* = 0.001), Medically Assisted Reproduction (*χ*^2^ = 4.683; *p* = 0.030), Past Perinatal Psychiatric Disorders (*χ*^2^ = 9.882; *p* = 0.002), on ISQ at T0 (*χ*^2^ = 145.953; *p* < 0.001), and ISQ at T1 (*χ*^2^ = 7.528; *p* = 0.006). Table 1 presents descriptive statistics, including all characteristics considered in both samples.

### 5.2. Criterion Validity of the ISQ

The criterion validity of the ISQ was assessed by examining the diagnostic accuracy of ISQ (antenatal period—T0) outcomes referenced to dichotomized PSQI scores (antenatal period—T0) (Table 2).

The ISQ score (antenatal period—T0) was significantly correlated with all the scales employed (antenatal period—T0), which are indicative of adequate convergent and discriminant validity (Table 3).

### 5.3. Reliability Statistic and Test–Retest Reliability

The entire ISQ scale showed excellent reliability with a Cronbach’s alpha of 0.92. The φ correlation coefficients were calculated to assess the test–retest reliability of the ISQ for a subsample of participants (*N* = 49) who completed the ISQ antenatally (T0) and postnatally (6-months postdelivery; T1). The correlation for the ISQ scores was 0.491, *p* < 0.001.

#### Screening Accuracy of the ISQ

In line with the formulated hypothesis, the ISQ offers a good diagnostic accuracy within the collected sample, it correctly identified 80% of cases identified by the DSM-5. The ISQ total score showed a diagnostic accuracy of 93% with a sensitivity of 79.5%, a specificity of 94.9%, a positive predictive power (PPP) of 70.5%, and a negative predictive power (NPP) of 96.8% (Table 4).

**Figure 1:**
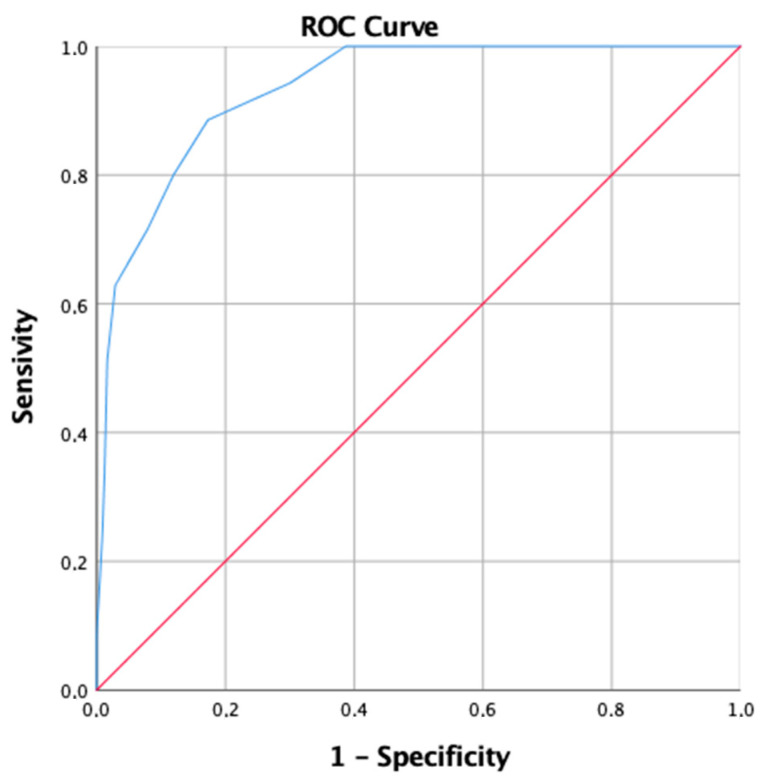
Graphical representation of receiver operator characteristic curve of the PSQI global score.

Furthermore, PSQI global score showed a high diagnostic accuracy within the collected sample, with an AUC value of 0.934 (*SE* = 0.018) (Figure 1). Through the ROC curve, it is also possible to identify the *best cutoff*, i.e., the value of the test that maximizes the difference between true positives and false positives (Youden’s index) [49]. In our case, the best cutoff for the PSQI is 8.5 which is associated with a sensitivity of 89% and a false positive rate of 17%.

## 6. Discussion

Sleep disorders in pregnant women and new mothers are frequent. They may enhance distress levels [11] and expose them to postpartum depression [18]. Perinatal depression has a deep impact on mothers and their partners with significant consequences on the infant that includes increased risk for low birth weight and prematurity, impairment on the interaction between mother and child, infant malnutrition during the first year of life, as well as on the cognitive and emotional development of the child [50,51]. Suicidal behavior is the main maternal complication of perinatal depression and is the second most common cause of mortality in postpartum women [52]. Hence, there is a need to assess and monitor insomnia during the perinatal period. Specific instruments to rate insomnia during this period are few; the Insomnia Symptom Questionnaire [31] is short and easy-to-use, and validated to identify insomnia in late pregnancy but had not received heretofore validation in Italian. Our study investigated the psychometric properties of the Italian Version of the ISQ in pregnant women with and without DSM-5 insomnia disorder, investigating the validity, reliability, and convergent and discriminant validity of the tool during late pregnancy and 6-months postpartum according to the DSM-5 criteria. We used the Italian version of the ISQ and also investigated a set of variables that included assessments of temperament, depression, anxiety, and hypomania.

In agreement with the literature [53], results showed that women with psychiatric family history, past perinatal psychiatric disorders, and those with past and current psychopharmacological treatment, exhibited more sleep impairments throughout pregnancy and postpartum compared to women without any history of psychiatric disorder. Additionally, women with more sleep disturbances in mid and late pregnancy showed more depressive and anxiety symptoms than women with fewer sleep disturbances [54,55,56], and significantly higher mean score on HCL-32, indicating bipolar disorder/diathesis [57]. As reported in the literature and confirmed by our samples, anxious, cyclothymic, depressive, and irritable affective temperaments were related to more dysfunctional sleep patterns [12,58,59]. Further, it is not surprising that significantly more women undergoing assisted reproductive treatment were in our Insomnia Sample. Short sleep duration, excessive daytime sleepiness, and poor sleep quality are common in women undergoing in vitro fertilization (IVF), and sleep duration may be a mediator of important markers of IVF success [60].

The findings provided evidence for the very high internal consistency of the Italian version of ISQ (Cronbach’s alpha of 0.92) with a better reliability coefficient compared to the original version [31]. Results also showed that the Italian version of the ISQ correlates strongly with established screening instruments, known to be sensitive to clinical insomnia such as the PSQI. The PSQI is used for subjective assessment of sleep quality and for identifying good and bad sleepers. However, it was not designed to assess insomnia based on diagnostic criteria or to investigate insomnia in the perinatal period. PSQI reliability and validity for identifying people who have difficulty initiating or maintaining sleep, necessary symptoms for the diagnosis of insomnia, may be improved by using a more stringent cutoff score than suggested (>5) [27,31,61,62]. Our results indicate that the best PSQI cutoff score to assess insomnia is 8.5 with a sensitivity of 89% and a false positive rate of 17%. In addition, the ISQ final score was significantly correlated with all the scales employed to investigate depression, anxiety, and hypomania, which indicates adequate convergent and discriminant validity.

The utility of this tool in pregnancy may be to identify women with persistent severe sleep problems. This may be clinically relevant given the emerging evidence that sleep disturbance increases the risk for adverse pregnancy outcomes.

It is curious that in our sample, only 44 pregnant women out of the final 292 included (15.07%) had insomnia. The literature generally reports higher prevalence rates in the third trimester of pregnancy, the time of our assessment, from 39.7% [9] to 42.4% [63]. It is possible that the different diagnostic methods used account for the discrepancy in findings.

## 7. Conclusions

Even though polysomnography is the most objective method for the assessment of most sleep disorders, the ICSD-3 and DSM-5 do not recommend it for the diagnosis of insomnia disorder due to its low feasibility. Subjective measures of sleep are a widespread issue in sleep research; however, daily fluctuations of sleep are hardly described by self-report questionnaires, such as the ISQ, which aims to investigate sleep quality over the past month. Furthermore, self-report estimates are very vulnerable to recall bias and overt or covert tendency to exaggerate the number and severity of symptoms [64]. A further possible limitation of the study could be related to the relatively small sample size to assess the test-retest reliability of the ISQ in a subsample of participants (*N* = 49). The fact that the original ISQ focused on the DSM-IV-TR, while we used the DSM-5, did not affect our results, inasmuch as the two diagnostic versions do not substantially modify insomnia diagnosis.

Future research should focus on the impact of maternal insomnia, as assessed through the ISQ, on future parenting style and child development, so as to identify methods to reduce it and ensure good maternal sleep in the perinatal period [65].

In conclusion, the results of the present study indicate that the ISQ is a useful, valid, and reliable tool for the assessment of perinatal insomnia also in the Italian language. The Italian version appears to be equivalent to the original version and to provide good and reliable discrimination between normal and pathological groups. The tool could be easily administered by obstetrics staff in everyday clinical practice.

## Figures and Tables

**Table 1:** Descriptive statistics of No-Insomnia and Insomnia samples.

	No-Insomnia Sample *N* (%)	Insomnia Sample *N* (%)		*df*	*p*
**Continuous Variables**			***F***		
**Age**					
*M*(*SD*)	33.13 (5.11)	34.10 (4.60)	1.254	1/289	0.264
*Min-Max*	19–46	22–42			
**BMI Early Pregnancy**					
*M*(*SD*)	22.59 (3.81)	22.92 (3.02)	0.197	1/245	0.658
*Min-Max*	15.63–42.52	17.44–28.98			
**TEMPS-A Depressive**					
*M*(*SD*)	5.49 (2.38)	8.70 (4.06)	**41.855**	**1/248**	**<0.001**
**TEMPS-A Cyclothymic**					
*M*(*SD*)	3.07 (2.94)	5.64 (3.66)	**20.226**	**1/248**	**<0.001**
**TEMPS-A Hyperthymic**					
*M*(*SD*)	10.73 (3.76)	9.73 (4.80)	1.889	1/245	0.171
**TEMPS-A Irritable**					
*M*(*SD*)	1.88 (2.21)	4.15 (3.29)	**26.140**	**1/242**	**<0.001**
**TEMPS-A Anxious**					
*M*(*SD*)	5.01 (4.04)	10.27 (5.35)	**43.933**	**1/242**	**<0.001**
**EPDS**					
*M*(*SD*)	5.26 (3.77)	10.46 (2.61)	**52.439**	**1/289**	**<0.001**
**SAS**					
*M*(*SD*)	33.51 (6.52)	39.94 (5.69)	**31.315**	**1/271**	**<0.001**
**HCL-32**					
*M*(*SD*)	11.68 (6.56)	15.43 (6.58)	**8.674**	**1/254**	**0.004**
**PSQI Global Score**					
*M*(*SD*)	5.75 (3.02)	12. 29 (2.89)	**144.850**	**1/276**	**<0.001**
**Categorical Variables**			**Chi-squared test**		
**Level of Education**			0.691	2	0.708
Middle school	14 (5.6)	1 (2.6)			
High school	101 (40.1)	17 (43.6)			
University/Postgraduate	137 (54.4)	21 (53.8)			
N/A	1	-			
**Occupation**			1.512	1	0.219
Unemployed	55 (21.8)	5 (13.2)			
Employed	197 (78.2)	33 (86.8)			
N/A	1	1			
**Partner**			0.474	1	0.491
No	3 (1.2)	0 (0)			
Yes	240 (98.8)	38 (100)			
N/A	10	1			
**BMI cutoff**			2.533	3	0.469
Underweight	20 (9.1)	2 (7.4)			
Normal weight	152 (69.1)	17 (63.0)			
Overweight	41 (18.6)	8 (29.6)			
Obese	7 (3.2)	0 (0)			
N/A	33	12			
**BMI—Range Weight Gain**			0.389	2	0.823
Under range	91 (44.0)	9 (39.1)			
Normal	81 (39.1)	9 (39.1)			
Above range	35 (16.9)	5 (21.7)			
N/A	46	16			
**Medical Conditions**			0.020	1	0.888
No	192 (75.9)	30 (76.9)			
Yes	61 (24.1)	9 (23.1)			
**Psychiatric History**			3.068	1	0.080
No	205 (81.3)	27 (69.2)			
Yes	47 (18.7)	12 (30.8)			
N/A	1	-			
**Previous Psychopharmacological Therapy**			**16.772**	**1**	**<0.001**
No	238 (94.1)	29 (74.4)			
Yes	15 (5.9)	10 (25.6)			
**Current Psychopharmacological Therapy**			0.058	1	0.810
No	248 (98.0)	38 (97.4)			
Yes	5 (2.0)	1 (2.6)			
**Psychiatric Family History**			**10.287**	**1**	**0.001**
No	192 (75.9)	20 (51.3)			
Yes	61 (24.1)	19 (48.7)			
**Menstrual Cycle Regularity**			0.446	1	0.504
No	49 (19.6)	9 (24.3)			
Yes	201 (80.4)	28 (75.7)			
N/A	4	2			
**Premenstrual Syndrome**			3.199	1	0.074
No	137 (55.0)	15 (39.5)			
Yes	112 (45.0)	23 (60.5)			
N/A	4	1			
**Others Completed Pregnancies**			0.118	1	0.731
No	156 (62.4)	22 (59.5)			
Yes	94 (37.6)	15 (40.5)			
N/A	3	2			
**Abortions**			1.746	1	0.186
No	181 (72.7)	23 (62.2)			
Yes	68 (27.3)	14 (37.8)			
N/A	4	2			
**Caffeine**			1.576	1	0.209
No	126 (50.4)	15 (39.5)			
Yes	124 (49.6)	23 (60.5)			
N/A	3	1			
**Tobacco**			0.053	1	0.818
No	221 (90.2)	32 (91.4)			
Yes	24 (8.6)	3 (8.6)			
N/A	8	4			
**Alcohol**			0.202	1	0.653
No	245 (98.4)	37 (97.4)			
Yes	4 (1.6)	1 (2.6)			
N/A	4	1			
**Narcotic Substances**			1.073	1	0.300
No	248 (99.2)	37 (97.4)			
Yes	2 (0.8)	1 (2.6)			
N/A	3	1			
**Assisted Fertilization**			**4.683**	**1**	**0.030**
No	209 (83.9)	36 (97.3)			
Yes	40 (16.1)	1 (2.7)			
N/A	4	2			
**Past Perinatal Psychiatric Disorders**			**9.882**	**1**	**0.002**
No	236 (93.7)	29 (78.4)			
Yes	16 (6.3)	8 (21.6)			
N/A	1	2			
**Pregnancy Complications**			0.052	1	0.819
No	183 (73.2)	27 (75.0)			
Yes	67 (26.8)	9 (25.0)			
N/A	3	3			
**Rest period**			0.003	1	0.955
No	191 (76.1)	28 (75.7)			
Yes	60 (23.9)	9 (24.3)			
N/A	2	12			
**Hospitalization During Pregnancy**			0.767	1	0.381
No	237 (93.7)	36 (97.3)			
Yes	16 (6.3)	1 (2.7)			
N/A	-	2			
**Partner’s Support**			1.526	1	0.217
No	14 (5.6)	4 (10.8)			
Yes	238 (94.4)	33 (89.2)			
N/A	1	2			
**Family’s Support**			2.604	1	0.107
No	52 (20.6)	12 (32.4)			
Yes	200 (79.4)	25 (67.6)			
N/A	1	2			
**Stressful Events**			0.065	1	0.799
No	136 (54.8)	20 (52.6)			
Yes	112 (45.2)	18 (47.4)			
N/A	5	1			
**ISQ—T0**			**145.953**	**1**	**<0.001**
**No**	**240 (94.9)**	**8 (20.5)**			
**Yes**	**13 (5.1)**	**31 (70.5)**			
**ISQ—T1**			**7.528**	**1**	**0.006**
**No**	**42 (93.3)**	**2 (50.0)**			
**Yes**	**3 (6.7)**	**2 (50.0)**			

*Note*. N/A: data not available.

**Table 2:** Cross-tabulations between PSQI global score (cut-off of >5 and >10) and ISQ (T0) classifications of subjects (No-Insomnia and Insomnia).

	PSQI Global Score		PSQI Global Score		Total
	≤5	>5	≤10	>10	
**ISQ**					
**No-Insomnia**	124	122	216	20	236
**Insomnia**	0	42	18	24	42
**Score _(Yes or No)_**	124	154	234	44	278

PSQI global score > 5: *χ*^2^ = 39.837 *p* < 0.001. PSQI global score > 10: *χ*^2^ = 63.391 *p* < 0.001.

**Table 3:** Correlations matrix between the ISQ (T0) total score and other scales (T0).

		ISQ	PSQI	SAS	EPDS	HCL-32
**ISQ**	*r_pb_*	-	0.519	0.352	0.366	0.167
	*p*		**<0.001**	**<0.001**	**<0.001**	**0.007**
	*N*	292	278	273	291	256

Abbreviations: EPDS: Edinburgh Postnatal Depression Scale; HCL-32: Hypomania CheckList-32; ISQ: Insomnia Symptom Questionnaire; PSQI: Pittsburgh Sleep Quality Index; SAS: Zung Self-rating Anxiety Scale.

**Table 4:** Cross-tabulations between DSM-5 diagnosis and ISQ classifications of subjects (No-Insomnia and Insomnia).

	DSM-5 Diagnosis		Total
	Insomnia	No-Insomnia	
ISQ			
Insomnia	31	13	44
No-Insomnia	8	240	248
Total	39	253	292

*χ*^2^ = 145.95; *p* ≤ 0.001 Cramér’s V = 0.707, *p* ≤ 0.001.

## Data Availability

The dataset used and analyzed in the current study is available from the corresponding author upon reasonable request.
